# The impacts of garlic and voluntary training alone or together on myocardial miR-126 and miR-210 gene expressions and angiogenesis in healthy rats

**DOI:** 10.34172/jcvtr.2020.33

**Published:** 2020-08-31

**Authors:** Rafighe Ghiasi, Alireza Alihemmati, Roya Naderi

**Affiliations:** ^1^Drug Applied Research Center of Tabriz University of Medical Sciences, Tabriz, Iran; ^2^Department of Histology and Embryology, Faculty of Medicine, Tabriz University of Medical Sciences, Tabriz, Iran; ^3^Nephrology and Kidney Transplant Research Center, Urmia University of Medical Sciences, Urmia, Iran; ^4^Department of Physiology, Faculty of Medicine, Urmia University of Medical Sciences, Urmia, Iran

**Keywords:** Voluntary Exercise, Garlic, miR, Cardiac Angiogenesis

## Abstract

***Introduction:*** microRNAs (miRs) play a critical role in both physiological and pathological processes. Recent studies have shown that garlic and exercise training have many beneficial effects in different disorders including cardiovascular disease. However, their mechanisms have not been fully understood. This study sought to investigate the impact of garlic and voluntary training alone or together on themiR-126 and miR-210 gene expressions and cardiac angiogenesis.

***Methods:*** Male Wistar rats were divided into four groups (n=7): (1) Control, (2) Garlic, (3) Exercise, and (4) Garlic+ Exercise. Animals were gavaged with raw fresh garlic homogenate (250 mg/kg body weight/day) or were subjected to voluntary training alone or together for about 6 weeks. The expressions of miR-126 and miR-210 in the heart tissue were measured by real-time PCR and lipid profile in serum was assessed by enzymatic kits. Angiogenesis was determined by immuno staining detection of PECAM-1 and CD31 in the heart tissue.

***Results:*** Garlic and exercise up-regulated myocardial miR-126 (*P* < 0.01), miR-210 (*P* < 0.001)expressions, and angiogenesis (*P* < 0.001) which was evidenced by higher CD31 expression. Besides, combination of garlic and exercise amplified their effects on those parameters (*P* < 0.001). Moreover, both voluntary exercise and garlic alone (*P* < 0.01) or together (*P* < 0.001) markedly modulated serum lipid profile.

***Conclusion:*** Voluntary exercise and garlic treatment for 6 weeks enhanced myocardial angiogenesis. These alterations were partly due to the increment of miR-126 and miR-210 expressions in the heart tissue in relation to improvement in lipid profile.

## Introduction

microRNAs (miRs) are known as a class of small noncoding RNAs which involve in the modulation of gene expression at RNA levels. During the last decades, growing evidence has been reported that miRs play important regulatory roles in many biological processes^1^ and also, are assumed promising preventive and therapeutic targets for several diseases including cardiovascular disorders.^2^ Several studies have indicated that miRs could have been introduced as important modulators of angiogenesis.^3^

So it was assumed that the angiogenic response of the vascular endothelium is regulated by miRs, suggesting a new window in angiogenesis phenomenon.^4^

Among them, miR-126 and miR-210 are important regulators, involved in neovasculogenesis. It was known that miR-126 was considered as a pivotal endothelial-specific miR, involved in heart angiogenesis via vascular endothelial growth factor (VEGF) pathway as an important angiogenic factor.^3^ Also, recent studies have focused on the role of miR-210 in angiogenesis process.^5^ miR-210, a master hypoxamir, induces an angiogenic response in endothelial cells and enhances microcirculation under both physiological and pathophysiological situations.^5^ Exercise training is a good strategy for the cardiovascular system in part through improved lipid profile, increased blood flow in vessels by structural changes in vasoreactivity of the coronary arteries.^3^ Even so, Da Silva et al has been demonstrated that swimming training promoted angiogenesis in the heart of rats.^3^

In literature, there is some evidence about anti-inﬂammatory, antioxidant, antiviral, anti-cancer properties of garlic in several organs.^6^ Garlic provides considerable health benefit that was known as a natural component for the prevention and treatment of several disorders including cardiovascular disease. It decreased the risk of ischemic stroke through mitigating platelet aggregation, dyslipidemia, and hypertension.^7^ Previously, Chiang et al exhibited that garlic ingestion enhanced neovasculogenesis in human endothelial progenitor cells to prevent ischemic injuries.^8^ Moreover, our groups documented the angiogenic effect of garlic in diabetic heart.^9^

On the other hand, the effect of garlic on inhibition neovasculogenesis for protecting organs and reducing tumor burden has been mentioned.^10,11^ Matsurra et al declared that aged garlic extract could inhibit tumor growth by blocking angiogenesis through the suppression of proliferation, migration, and tube formation.^12^ Based on the mentioned points angiogenesis response occurs in physiological and pathological conditions. To our knowledge, no study was designed focusing on the effect of garlic on the angiogenesis in the healthy heart tissue.

Dyslipidemia is probably associated with the changes of liver X receptor α expression in some tissues including liver and intestine and also stimulation of NADP which is contributing to eNOS inhibition and impaired angiogenesis.^9^

Therefore, dyslipidemia may lead to endothelial dysfunction by decreased nitric oxide synthesis and increased reactive oxygen species (ROS) synthesis, resulting in the development of coronary heart disease and cardiac events through angiogenesis disturbances.^13^ In this regard lipoproteins as a strong independent predictor of cardiovascular disease has an impact on miR expression in endothelial cells.^14^ It was documented that, the miR-126 level is in association with LDL ( low-density lipoprotein) in which increased LDL level results in down-regulation of miR-126 and decreased vasculogenesis.^15^

To the best of our knowledge, considering the above-mentioned contents, there are no studies at present reporting the combination impact of garlic and voluntary training on miRs involved in cardiac angiogenesis. Thus, the purpose of this study was to examine, for the first time, the role of garlic and voluntary training alone or in combination on both miR-126 and miR-210 levels and also lipid profile in relation to cardiac angiogenesis in healthy rats.

## Materials and Methods

### Animals and experimental design

All animal care and experimental procedure were conducted by specific board of medical ethics in Tabriz University of Medical Sciences and was done in an ethical manner according to the doctrines of Laboratory Animal welfare. The animals were kept in a room under environmental controlled conditions including temperature (24±2°C) and light–dark cycle (12-h) and also allowed free access to commercially standard regular rat normal diet for 24 h per day, including (1) carbohydrate 60% (w/w), (2) fat 2% (w/w), (3) protein 17.5% (w/w), and (4) fiber 8% (w/w), and (5) water *ad libitum* . Twenty-eight male Wistar rats 3-4 months of age and weighing 200-250 gram^16^ were randomly divided into four groups (n= 7): (1) Control, (2) Garlic, (3) Exercise and (4) Garlic+Exercise. In the current study sample size was defined based on our similar previous investigations.^9,17,18^

### Voluntary exercise

The running-wheel apparatus and it’s set up for voluntary training (1.00 m circumference, Tajhiz Gostar) was the same as explained according to our previous study.^19^ Briefly, the rats were permitted free access to the wheel for 6 weeks and the wheel revolutions were recorded daily. The animals excluded from the study, in the case that the running distance was lower than 2000 m/day.

### Preparing garlic homogenate

Fresh garlic homogenate was prepared from garlic bulbs. For this purpose, they were peeled off, cut into small pieces, and then ground into a paste. Afterward, garlic preparation dissolved in distilled water and gavaged orally each day. The homogenate was prepared freshly every morning.

### Sampling

At the end of the 6th week, rats were deeply euthanized with ketamine (60 mg/kg) and xylazine (6 mg/kg),^20,21^ blood was drawn from all animals by cardiac puncture for measurement of lipid profile.

Then the hearts were quickly isolated and the left ventricles were excised, frozen in liquid nitrogen, and stored in deep freeze (-80°C) for later measurements. Also, a piece of each left ventricle fixed at 10% buffered formalin solution. Myocardial tissue was used for miR extraction and real-time PCR study and evaluation of angiogenesis.

### miR extraction and real-time PCR

miR was extracted from the heart tissue using a miRCURYTMRNA special isolation kit (Exiqon, Vedbaek, Denmark) in accordance with the manufacturer’s instructions.^22-24^ The procedure was carried out based on the spin column by a proprietary resin as a separation matrix for RNA from other cell ingredients. RNA content and purity was assessed with Nanodrop 1000 spectrophotometer apparatus (Thermo scientific, Wilmington, DE 19810 USA). The expression profile of miR-126 was performed on total RNA extracts by the universal cDNA synthesis kit. In brief, total RNA with miRs was polyadenylated then, cDNA was synthesized by a poly (T) primer with a 3 degenerate anchor and also, a 5 universal tag (Exiqon, Vedbaek, Denmark). obtained cDNA was utilized as a template for miRs quantitative real-time PCR by using materials namely SYBR Green master mix in our labratory (Exiqon, Vedbaek, Denmark). LNA (Locked Nucleic Acid) a novel artificial nucleotide analog primer (Exiqon, Vedbaek, Denmark) for miRs has been listed in Table 1. Real-time PCR reaction was done on a Bio-Rad iQ5 detection device (Bio-Rad, Richmond, CA, USA). The obtained PCR outputs were normalized with housekeeping gene including rno-miR-191.^23^ The 2-^ΔΔCt^ method was used to evaluate relative quantitative contents of miR-126 and miR-210.^25^ The data were displayed as the fold-difference to the relevant controls.

**Table 1:** Target sequence list for miRs

**Gene name**	**Accession number**	Target sequence*
rno-miR-191	MIMAT0000440	CAACGGAAUCCCAAAAGCAGCUG
hsa-miR-126	MIMAT0002957	UCGUACCGUGAGUAAUAAUGC
dme-miR-210	MIMAT0001233	UUGUGCGUGUGACAGCGGCUA

* Sequences were obtained from miRBase (http://www.mirbase.org/).

### Immunostaining for PECAM-1/ CD31

Angiogenesis evaluation in the myocardium was assayed by PECAM-1/ CD31. For this purpose, a transversal section of the left ventricles was excised and fixed at 10% formalin buffer, dehydrated in various ascending grades of ethanol baths then, kept the samples in paraffin. Serial 3µm thick sections were provided and stayed on the charged glass surface according to standard histological protocols. Tissue specimen were deparaffinized by using xylene and dehydrated in a graded series of ethanol. Slides were incubated sequentially with proteinase K and treated by 0.3% hydrogen peroxide for blocking endogenous peroxidase activity. Sections were laminated by primary antibody CD31 (Santa Cruz, USA) a marker of angiogenesis, and incubated at +4°C overnight. Then, tissue sections washed and then incubated with standard avidin–biotin complex reagent (ABC; Santa Cruz) in accordance with protocol. DAB (Diamino-benzidine, Santa Cruz), was used for incubation the slides as the chromagen, and counterstained with Mayer’s hematoxylin. Ultimately, sections were cleared through xylene, mounted with Entellan and then observed by a precise light microscope.

### Assessment of immunostaining

Three to 5 sections of 1 mm^2^ space were obtained randomly at magnification 400×, for quantification of immunostaining. Staining intensity and also, the number of positive cells was counted semi-quantitatively. So, the intensity scoring for endothelial marker, CD31 was detected within each area at 400× magnification. CD31 positive structures were calculated for 5 to 6 slides per rat and 10 fields per slide.

For investigation of immunostaining as an indicator of capillary blood vessels, the granulation tissue was considered as a positive control, and the scores of the staining intensity was as follows: 0 (<10%); 1 (10% to 25%); 2 (25% to 50%); 3 (50% to 75%) or 4 (75% to 100%).^9^

### Lipid profile measurement

Blood samples were collected by cardiac puncture. The samples centrifuged at a speed of 3500 rpm at 4°C for 10 min and then, serum was isolated. Triglyceride levels were assessed by special enzymatic kits (ZiestChem Diagnostic kits, made in Iran) by glycerol as a standard. Furthermore, high- density lipoprotein (HDL) and also LDL levels were detected by quantitative enzymatic colorimetric method by using special diagnostic kits, (ZiestChem, Iran) considering cholesterol as the standard. All reactions were evaluated by Digital UV/VIS spectrophotometer apparatus (CE 292, series 2, Cecil Instruments, Cambridge, England).

### Statistical analysis

Normal distribution of our data was identified using Kolmogorov–Smirnov test. Statistical analyses were done using SPSS 16.0 for Windows (SPSS, IBM, Chicago, USA). The statistical differences between the groups were assessed by using one-way ANOVA and Tukey’s post hoc test. The data are presented as the mean±SEM, and *P* < 0.05 is considered as statistically significant.

## Results

### Impacts of garlic and voluntary training on miR-126 gene expression in the myocardium

**Figure 1:**
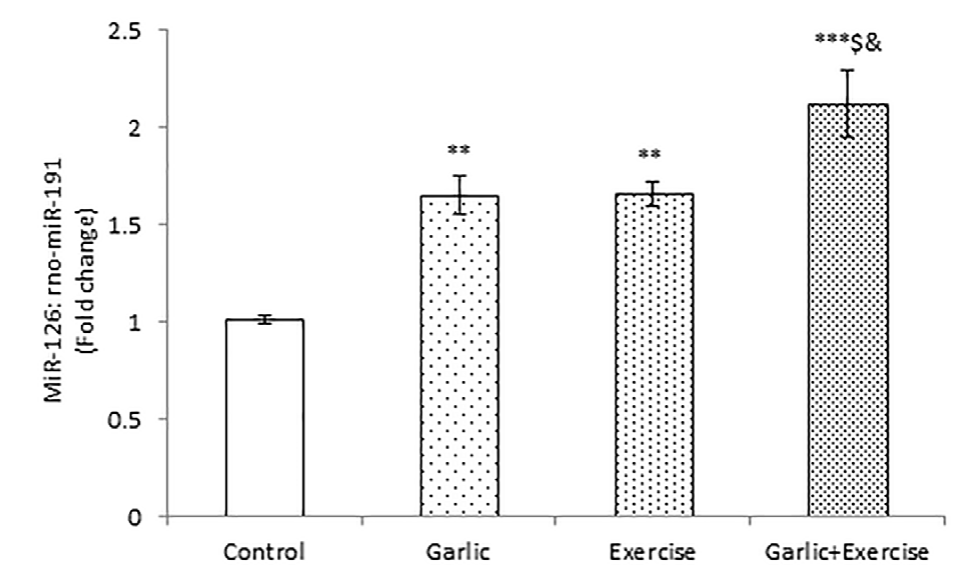
Real-time quantitative PCR data of miR-126 gene expression in the heart tissue of different groups. The values represent means±S.E.M for study groups. ** *P* < 0.01 and ***P < 0.001 compared with control group, ^$^*P* < <0.05 compared with Garlic group, ^&^*P* < 0.05 compared with Exercise group

As shown in Figure 1, the myocardial miR-126 expression level was significantly increased in Garlic and Exercise groups alone (*P* < 0.01) and in combination (*P* < 0.001) in comparison to the Control group. Moreover, treatment with Garlic and performing voluntary exercise together demonstrated significantly (*P* < 0.05) higher level of miR-126 gene expression compared with garlic treatment and voluntary exercise alone.

### Impacts of garlic and voluntary training on miR-210 gene expression in the myocardium

**Figure 2:**
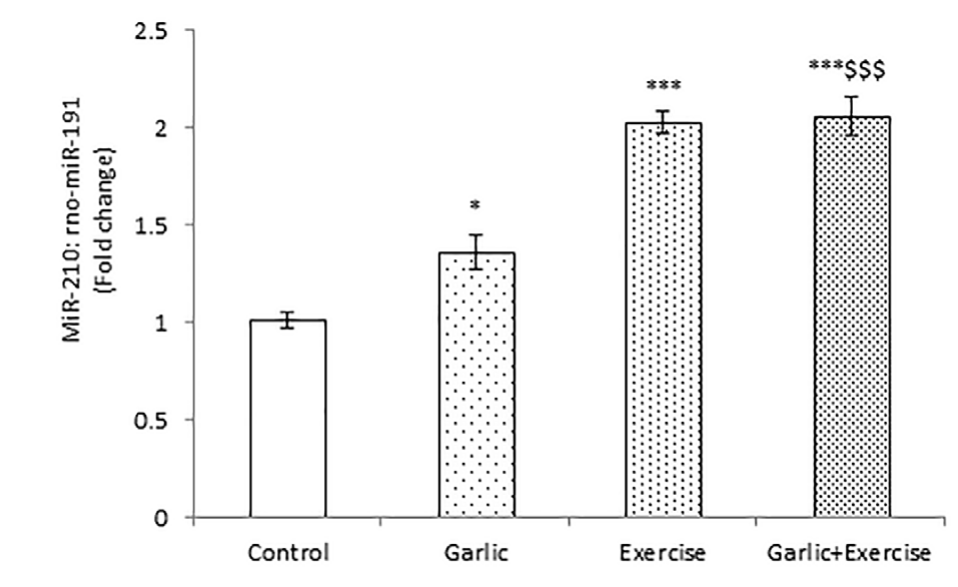
Real-time quantitative PCR data of miR-210 gene expression in the heart tissue of different groups. The values represent means ± SEM for study groups. * *P* < 0.05 and *** *P* < 0.001 compared with control group, ^$$$^*P* < 0.001 compared with Garlic group

As shown in Figure 2, garlic (*P* < 0.05) and voluntary exercise alone or in combination (*P* < 0.001) significantly increased the expression of miR-210 compared with the control. Treatment with both Garlic and voluntary exercise significantly enhanced miR-210 gene expression compared to the Garlic group (*P* < 0.001).

### Impacts of garlic and voluntary training on angiogenesis in the myocardium

**Figure 3:**
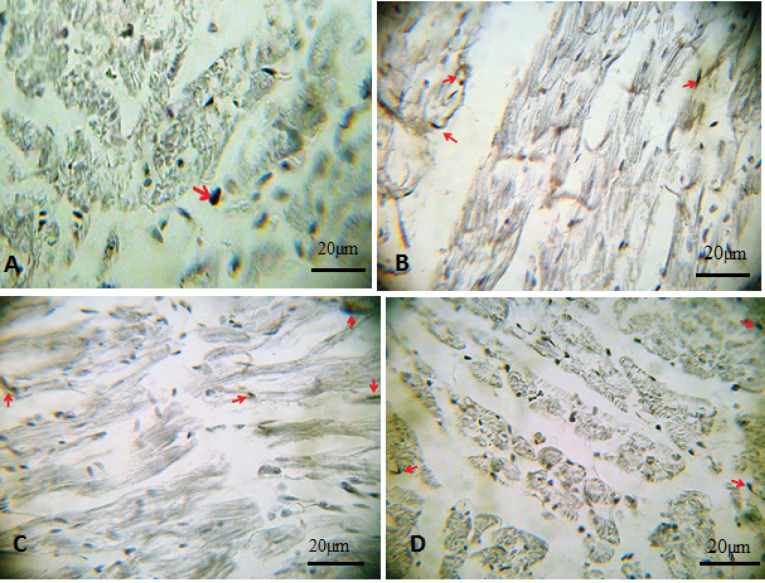
Immunohistochemical detection of CD31 in myocardial vessels of different groups. Brown stained tissues demonstrating CD-31 immunostained endothelial cells in groups including (A): Control, (B): Garlic, (C): Exercise, (D): Garlic+Exercise. The intensity of reaction for CD31 (arrow head) increased in the Garlic and Exercise group compared to the control group. The combination effect of Garlic treatment and Exercise training-induced more angiogenesis compared with garlic and exercise alone (400x magnification). Scale bars are as indicated

**Figure 4:**
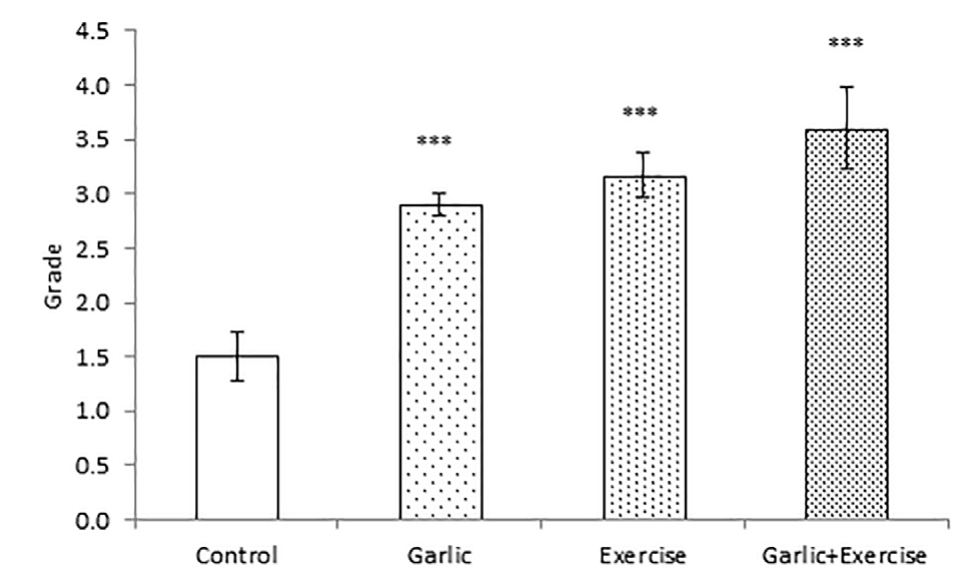
Impacts of garlic and voluntary training on angiogenesis in study groups. The intensity of the staining was classified into four categories: 0 (<10%)- 1 (10-25%)- 2 (25-50%)-3 (50 -75%) and finally 4 (75-100%). The values represent means ± SEM for study groups. ***P < 0.001 compared with Control group

To appraise angiogenesis in the obtained transversal section of the ventricles, expression of CD31 antigen was investigated by immunohistochemistry staining technique. Brown stained tissues indicate CD-31 immunostained endothelial cells (Figure 3). In Figure 4, we demonstrated the intensity of the staining that was scored as follows: 0 (<10%), 1 (10% to 25%), 2 (25% to 50%), 3 (50% to 75%) and 4 (75% to 100%). According to our findings, six weeks of garlic treatment, performing the voluntary exercise, or their combination significantly (*P* < 0.001) increased angiogenesis in their left ventricle in comparison to the Control group (Figures 3 and 4). Combination of garlic consumption and performing voluntary exercise-induced more angiogenesis compared with garlic and exercise alone; although it was not significant.

### Impacts of garlic and voluntary training on serum lipid profile

Table 2 summarizes the effect of garlic consumption and voluntary exercise performance on lipid profile. As shown in Table 2, garlic and voluntary exercise alone (*P* < 0.05) or in combination (*P* < 0.01) significantly reduced serum triglyceride levels in comparison with the Control group. In parallel, serum levels of LDL and HDL modulated following garlic and voluntary exercise alone (*P* < 0.01) or together (*P* < 0.001). Six weeks treatment with garlic and voluntary exercise alone or in combination significantly (*P* < 0.001) enhanced HDL/LDL ratio versus Control group. Furthermore, the ratio of HDL/LDL was significantly (*P* < 0.05) higher in the Garlic + Exercise group as compared with the Garlic group.

**Table 2:** Serum lipid profile in study groups after 6 weeks treatment (Mean ± SEM, n = 7)

**Variants**	**Control**	**Garlic**	**Exercise**	**Garlic+Exercise**
Triglyceride (mg/dL)	38.16±1.49	32.83±1.16*	32.5±1.14*	30.661±0.81**
LDL (mg/dL)	39±1.19	34.16±0.6**	33.66±0.55**	31.83 ±0.7***
HDL (mg/dL)	26.8±1.02	33.5±1.17**	35.16±0.47**	35.33±0.55***
HDL/LDL	0.68±0.04	0.98±0.03***	1.01±0.01***	1.1±0.03***$

* *P* < 0.05, ** *P* < 0.01 , *** *P* < 0.001 compared with control group and $P < 0.05 compared with Garlic group. Triglycerides = TG, High-density lipoprotein = HDL, Low-density lipoprotein = LDL.

## Discussion

The results of the current study indicated that treatment with garlic and performing voluntary exercise alone or in combination for 6 weeks, increased angiogenesis, the expressions of miR-126, and miR-210 in the rat myocardial tissue and also, ameliorated serum lipid profile. Moreover, we found that concomitant treatment of garlic and voluntary training had an additive result on the mentioned parameters.

It was reported that several miRs play a key role in regulating heart function that may lead to the discovery of the novel treatment strategy for vascular disorders.^9^ Even though, there are various miRs in the heart tissue, little evidence is available about the cardiac angiogenic response and special miRs that are involved in this process following garlic and voluntary exercise interventions. miR-126 is an endothelial-specific miR and it’s role in angiogenesis has been established.^26,27^ Previously, it was reported that vascular integrity and angiogenesis impaired when knockdown of miR-126 in animals was occurred.^26^ SPRED1 and PIK3R2 are targets of miR-126 for enhancing VEGF signalling in part by phosphorylation of ERK and AKT pathway,^28^ which are critical downstream components of the angiogenic pathways led to capillary formation. This event leads to vascular sprouting and angiogenesis.^26^ So, the elevated level of miRNA-126 may be associated with a reduction in the expression of it’s two targets, contributing to angiogenic response.

Our finding demonstrated that angiogenesis increased in the myocardium in response to garlic and voluntary exercise alone or in combination. In line with our results, studies have demonstrated that exercise training increased cardiac angiogenesis both under healthy^13^ and pathological conditions,^29,30^ which indicates an important role of physical activity as a non-pharmacological intervention of cardiovascular disorders. However, the precise mechanisms have not been fully defined. Little information confirmed that voluntary exercise could modulate miRs expression in the myocardial tissue. In this study, we found that voluntary exercise increased the expression of miR-126 in the healthy myocardium.

In agreement with our study, DA Silva et al showed that swimming training enhanced cardiac miR-126 expression in healthy rats, which was probably in association with exercise-induced cardiac angiogenesis.^3^ The previous report demonstrated that, the exercise showed an elevation of circulating miR-126 in healthy persons.^31^ Considering the above information, cardiac angiogenesis is in part contributed to exercise-induced miR-126 gene expression and VEGF up-regulation, which stimulates angiogenic pathways including the MAPK and PI3K/Akt/eNOS.^3^

Moreover, it was demonstrated that garlic usage in the diet is strongly associated with cardiovascular injury and ischemic insult. Cardioprotective effect of garlic has been attributed to the antioxidant activities,^19^ AMPK-regulated AKT/GSK-3β/HIF-1α pathway,^32^ and Akt-eNOS signaling pathways.^33^ However, it’s fully mechanisms is still in debate.

In this study, we identified that garlic treatment increased angiogenesis and miR-126 expression in the rat myocardium. Ejaz et al indicated that the ameliorated effect of aged garlic solution on wound healing was mediated by neovascularization.^34^ Also, garlic consumption increased angiogenesis in human endothelial progenitor cells contribute to protecting effect against ischemic injuries by suppressing the expression of miR-221.^8^ Studies reported that garlic extract induced angiogenesis partly by up-regulation of neovasculogenic c-kit protein level and augmentation of the PI3-K/Akt/NF-κB signaling pathways,^8^ which affects e-NOS activation and NO generation.^35^ Additionally, garlic may serve as a donor of hydrogen sulfide enzyme which has recently been recognized as an angiogenesis regulator.^36^ In turn, it was revealed that hydrogen sulfide could up-regulate miR-126 gene expression in various tissues.^37^

Therefore, it may be logical to suggest that increased hydrogen sulfide and corresponding increased miR-126 in garlic-treated rats might be one of the possible mechanisms of garlic-mediated increased angiogenesis.

miR-210 has been identified as a constant feature of the hypoxic response in various organs including cardiac muscle. Also, miR-210 modulates VEGF expression by targeting hypoxia-inducible factor 1-α (HIF-1α) leading to angiogenesis response via its target gene Ephrin-A3. It has been shown that myocardial angiogenesis enhanced via the up-regulation of miR-210 and VEGF in acute myocardial infarction following Huoxue Anxin Recipe.^38^

Recently it was demonstrated that plasma levels of miR-210 down-regulated in chronic kidney disease in response to acute exercise.^39^ On the contrary, various studies have demonstrated that miR-210 was not affected due to acute, exhaustive exercise, continuous aerobic exercise training,^35,40^ and swimming^2^ in the heart tissue. Also, in this regard, circulating miR-210 was reduced following 3-months basketball training in competitive male basketball champions.^1^ Despite the inconsistencies, in the current study, we showed that both garlic and voluntary exercise and their combination increased myocardial miR-210 level. It was indicated that exercise exerts local hypoxic conditions in the heart tissue leading to angiogenesis in a HIF-1α dependent manner. In line, it was reported that low Vo2max following exercise activity significantly increased miR-210 expression.^41^

Also, garlic protects the heart in ischemia-reperfusion injury in part by HIF-1α activation.^32^ Therefore, considering the above information, garlic can up-regulate the expression of miR-210 in the myocardium resulted in the angiogenesis process mediated by HIF-1α activation.

Angiogenesis impaired probably in order to dyslipidemia, which even happens in healthy people. It was possibly mediated through inhibition of eNOS activity because of the alteration of LXRα gene expression in some tissues including the liver and intestine augmentation of NADPH molecules causing impaired angiogenesis.,^42^ Also, dyslipidemia reduced levels of circulating miR-126.^26^ On the other hand, it was reported that exercise training ameliorated HDL-induced miR-126 expression.^14^ In the present study, intervention with garlic and voluntary raining alone or together with improved lipid profile in serum of healthy rats, that is in line with previous investigations.^11,43^ Also, the combined effect of garlic and voluntary training increased HDL/LDL in an additive manner, which used clinically for predicting the risk of cardiovascular disease.^44^ Based on the mentioned points, garlic and exercise training have possibly regulated angiogenesis in the myocardium through modulation of serum lipid profile and pro-angiogenic miRs expression. In relevance to our study limitation, we did not measure the other molecular mechanisms taking part in angiogenesis. Further studies are warranted to evaluate these effects in the future.

## Conclusion

In conclusion, the present study indicated that garlic and voluntary training enhanced myocardial angiogenesis, accompanied by increased expression of miR-126 and miR-210, and also improved serum lipid profile. Thus, garlic and voluntary exercise alone and in combination have been confirmed not only as a way to maintain a healthy lifestyle but also as a powerful and safe non-pharmacological recommendation for prevention of vascular disturbances.

## Competing interests

None declared.

## Acknowledgements

This article is a part of Doctoral thesis submitted by Roya Naderi, from Drug Applied Research Center, Tabriz University of Medical Sciences.

## Ethical approval

This study was approved by the Ethics board on Animal Experiments of the Tabriz University of Medical Sciences with the protocol number 91.4-2.4.

## Funding

This study was supported by Drug Applied Research Centre, Tabriz University of Medical Sciences, Tabriz, Iran.
